# A flow cytometry-based neutralization assay for simultaneous evaluation of blocking antibodies against SARS-CoV-2 variants

**DOI:** 10.3389/fimmu.2022.1014309

**Published:** 2022-11-24

**Authors:** Leire Egia-Mendikute, Alexandre Bosch, Endika Prieto-Fernández, Laura Vila-Vecilla, Samanta Romina Zanetti, So Young Lee, Borja Jiménez-Lasheras, Ana García del Río, Asier Antoñana-Vildosola, Ander de Blas, Paloma Velasco-Beltrán, Marina Serrano-Maciá, Paula Iruzubieta, Majid Mehrpouyan, Edward M. Goldberg, Scott J. Bornheimer, Nieves Embade, María L. Martínez-Chantar, Marcos López-Hoyos, José M. Mato, Óscar Millet, Asís Palazón

**Affiliations:** ^1^ Cancer Immunology and Immunotherapy Lab, Center for Cooperative Research in Biosciences (CIC bioGUNE), Basque Research and Technology Alliance (BRTA), Bizkaia Technology Park, Derio, Spain; ^2^ Liver Disease Lab, Center for Cooperative Research in Biosciences (CIC bioGUNE), Basque Research and Technology Alliance (BRTA), Bizkaia Technology Park, Derio, Spain; ^3^ Servicio Inmunología, Hospital Universitario Marqués de Valdecilla-IDIVAL, Cantabria, Spain; ^4^ BD Biosciences, San Jose, CA, United States; ^5^ Precision Medicine and Metabolism Lab, Center for Cooperative Research in Biosciences (CIC bioGUNE), Basque Research and Technology Alliance (BRTA), Bizkaia Technology Park, Derio, Spain; ^6^ Centro de Investigación Biomédica en Red de Enfermedades Hepáticas y Digestivas (CIBERehd), Instituto de Salud Carlos III, Madrid, Spain; ^7^ Ikerbasque, Basque Foundation for Science, Bizkaia, Spain

**Keywords:** SARS-CoV-2, COVID-19, antibodies, flow cytometry, neutralization, beads array

## Abstract

Vaccines against SARS-CoV-2 have alleviated infection rates, hospitalization and deaths associated with COVID-19. In order to monitor humoral immunity, several serology tests have been developed, but the recent emergence of variants of concern has revealed the need for assays that predict the neutralizing capacity of antibodies in a fast and adaptable manner. Sensitive and fast neutralization assays would allow a timely evaluation of immunity against emerging variants and support drug and vaccine discovery efforts. Here we describe a simple, fast, and cell-free multiplexed flow cytometry assay to interrogate the ability of antibodies to prevent the interaction of Angiotensin-converting enzyme 2 (ACE2) and the receptor binding domain (RBD) of the original Wuhan-1 SARS-CoV-2 strain and emerging variants simultaneously, as a surrogate neutralization assay. Using this method, we demonstrate that serum antibodies collected from representative individuals at different time-points during the pandemic present variable neutralizing activity against emerging variants, such as Omicron BA.1 and South African B.1.351. Importantly, antibodies present in samples collected during 2021, before the third dose of the vaccine was administered, do not confer complete neutralization against Omicron BA.1, as opposed to samples collected in 2022 which show significant neutralizing activity. The proposed approach has a comparable performance to other established surrogate methods such as cell-based assays using pseudotyped lentiviral particles expressing the spike of SARS-CoV-2, as demonstrated by the assessment of the blocking activity of therapeutic antibodies (i.e. Imdevimab) and serum samples. This method offers a scalable, cost effective and adaptable platform for the dynamic evaluation of antibody protection in affected populations against variants of SARS-CoV-2.

## Introduction

SARS-CoV-2 emerged in late 2019 in Wuhan, resulting in a worldwide pandemic (1). Initial research identified the spike as a key structural component of the virus. The spike interacts with the membrane exposed angiotensin converting enzyme 2 (ACE2) through its receptor-binding domain (RBD) (2), leading to the infection of host cells (3). For this reason, existing vaccination strategies exploit the Spike as immunogen with the aim of inducing RBD-specific neutralizing antibodies (4–6). Of note, antibodies that bind to the N-terminal domain of S2 instead of RBD can present neutralizing activity (7, 8).

Given the evolving nature of the RBD sequence, several variants of concern have emerged since 2019. The mutations present in these variants influence the binding affinity of RBD to ACE2 and can result in escape from existing humoral immunity against the original Wuhan-1 strain. As a result, existing vaccines can lose efficacy against certain variants (9). This is the case of Omicron BA.1, a variant that emerged in South Africa in late 2021 (10) that presents several mutations that affect key residues on the RBD, leading to escape from neutralizing antibodies (11, 12).

The development of more efficient serological surveillance and improved antigenic design of second-generation vaccines are ongoing efforts that will support the control of emerging pandemic threats. In this context, different approaches have been developed to detect, and sometimes quantify, SARS-CoV-2 specific antibodies. These immunoassays, mainly based on ELISA or rapid tests, can detect the presence of antibodies but are not able to predict their neutralizing capacity against the virus (13, 14). The gold standard approach to measure the neutralizing activity of antibodies is based on the use of native origin viruses and surrogate assays that employ pseudotyped lentiviral particles expressing the SARS-CoV-2 spike protein and target cells expressing human ACE2 (15, 16). These cell-based assays are time consuming, have limited multiplexing capability and are not practical in scalable and regulated environments.

Existing immunoassays based on fluorescent bead arrays (17) have demonstrated superior sensitivity and multiplexing capability for measuring antibodies against several viral antigens simultaneously (18, 19). Here, we have developed a novel multiplexed bead array for flow cytometry (nC19BA) that determines the neutralizing capacity against four different variants in a fast, easy, and replicable manner. Using this assay, we have been able to demonstrate the superior antibody neutralizing capacity against emerging variants after the third campaign of vaccination compared to the response attained by individuals in 2020-2021 period, i.e., when individuals presented only one or two vaccine doses or were seropositive as a result of natural infection prior to the emergence of Omicron BA.1.

## Material and methods

### Cell culture

HEK293T and HEK293T-ACE2 cells (kindly provided by June Ereño-Orbea from CIC bioGUNE and Dr. Jean-Philippe Julien from The Hospital for Sick Children Research Institute) were cultured in DMEM (41966-029, Gibco). Media was supplemented with 10% FBS (10270106, Thermo Fisher) and 1% Penicillin–Streptomycin (15140122, Thermo Fisher).

### Serum samples

Serum samples were divided in four cohorts, based on sample collection date: i) preCOVID cohort (30 serum samples, collected in 2018-2019), ii) COVID cohort (30 serum samples from PCR-positive individuals collected in 2020-2021), iii) pre-Omicron cohort (a representative subset of the population collected in late 2021, before the third dose was administered, n=30 serum samples) and iv) post-Omicron cohort (a representative subset of the population collected after the outbreak of Omicron and the administration of the third dose of the vaccine, n=30 serum samples). Serum samples corresponding to preCOVID and seropositive individuals were provided by the Basque Biobank (https://www.biobancovasco.org) after approval from the corresponding ethics committee (CEIC-E 20-26, 1-2016, CEIm-E PI+CES-BIOEF 2020-04 and PI219130). Some samples were obtained during the yearly medical check-up of the working population of the Basque Country in 2018-2019 in collaboration with Osarten Kooperatiba Elkartea from Mondragon Corporation (20). The COVID cohort corresponding to patients presenting COVID-19 symptomatology and diagnosed by PCR were obtained after written informed consent and approval by the Cantabria Ethics Committee (CEIm Code: 2020.167).

### Microbead coating

Custom-made Becton Dickinson (BD) functional cytometric bead array (CBA) beads were used in preparation of streptavidin beads as described in the Functional Bead Conjugation Buffer Set Instruction Manual (BD 558556). Briefly, 1 mg of SMCC-modified streptavidin per mL (in 50 mM sodium phosphate, 0.2 M sodium chloride, pH 7.4) was used for conjugation to CBA beads as described in the above protocol. The streptavidin beads were suspended in 1x PBS, 0.5% BSA, pH 7.2 before coating with biotinylated RBD proteins. Streptavidin microbeads were then coated with antigen as described before (18). Briefly, streptavidin coated microbeads were incubated with 11 μg/mL of biotinylated RBD proteins from the following SARS-CoV-2 variants: RBD Wuhan-1 (Acrobiosystems SPD-C82E9), RBD-B.1.351 (β, Acrobiosystems SPD-C82E5), RBD- B.1.617.1 (κ, Acrobiosystems SPD-C82Ec) and RBD-B.1.1.529 (ο, Acrobiosystems SPD-C82E4). Uncoated microbeads were used as negative controls. All microbeads were incubated with an excess of D-biotin to saturate the remaining free streptavidin sites.

### nC19BA assay

Antigen-coupled microbeads were added to LoBind 1.5 mL Eppendorf tubes (Eppendorf 525-0133) in a volume of 50 μL of PBS (Gibco 10270-106) containing a total of 5000-6000 microbeads. After centrifugation (1000 x*g* for 5 min), microbeads were resuspended with 100 µL of diluted serum samples (1:5 in PBS, 20 µl of serum sample in 80 µl of PBS) or serially diluted commercially available antibodies against S1: clone AM122 (Acrobiosystems) and Imdevimab (Ibian technologies, SRBDC4-MAB1), starting from a concentration of 20 mg/mL. Negative control samples were prepared in PBS with 5% FBS (Gibco 10270-106). After a 30-minute incubation (4°C protected from light), samples were washed three times with PBS. Human recombinant ACE2 with murine IgG1 (rhACE2-mIgG1, recombinant fusion protein of human ACE2 ectodomain fused with murine IgG1 Fc produced in HEK293T eukaryotic cells, SinoBiological 10108-H05H) was added at 0.18 mg/mL (diluted in 100 µL of PBS containing 5% FBS) and incubated for 30 minutes at 4°C protected from light. Three washes were performed, and microbeads were incubated with an anti-mouse IgG1-BV421 antibody (1:200 in 100 µL of PBS supplemented with 5% FBS) for 30 mins at 4°C. A final wash was performed, and microbeads were resuspended in 200 µL of PBS for acquisition. At least 600 events for each type of microbead were acquired in a FACSymphony flow cytometer (BD Biosciences) and geometric mean fluorescence intensities (gMFI) were obtained. Results were analysed using FlowJo version 10 (BD Biosciences). The neutralizing percentage corresponding to each sample against each variant was measured with the following formula: % neutralizing = ([gMFI sample − gMFI max neutralization]/[gMFI max binding − gMFI max neutralization]) × 100). The value of gMFI maximum neutralization corresponds to the signal obtained with microbeads in the absence rhACE2. The value of gMFI max binding corresponds to the signal obtained in the absence of serum samples or blocking antibodies.

### Pseudotyped viral particle production

Pseudotyped lentiviral particles were generated by transfecting HEK 293T cells as described before (21). Briefly, 5 million HEK293T cells were seeded 24h before transfection in a P100 plate. Cells were transfected with plasmids encoding for the lentiviral backbone containing ZsGreen under the control of the CMV promoter (NR-52520, 5.79 µg), the SARS-CoV-2 spike protein (NR-52514, 1.97 µg), HDM-Hgpm2 (NR-52517, 1.27 µg), pRC-CMV-Rev1b (NR-52519, 1.27 µg) and HDM-tat1b (NR-52518, 1.27 µg), using the jetPEI kit (101-10N, Polyplus-transfection). 48 h after transfection, viruses were harvested from the supernatant, filtered through a 0.45 µm filter (514-0063, VWR) and mixed with lentiX (631232, Takara) in a 3:1 proportion at 4°C overnight. Then, viruses were centrifuged at 1500xG for 45 minutes and the pellet was resuspended in 200 µL of DMEM, aliquoted and stored at -80°C.

### Titration of pseudotyped lentiviral particles and infection of HEK293T-ACE2 cells

Viral titration was performed in HEK293T-ACE2 cells as described before (21). Then, 50,000 cells were seeded in 96-well plates, cultured overnight, and incubated with pseudotyped lentiviral particles (MOI: 0.34) for 24 h in the presence of a serial dilution of anti-RBD monoclonal antibodies or serum samples (1:5 dilution in culture media). HEK293T-ACE2 cells were harvested and Zsgreen positive cells were quantified by flow cytometry using a FACSymphony (BD Biosciences). Results were analyzed in Flowjo (BD Biosciences).

### ELISA

96-well ELISA plates (Nunc Maxisorp 44-2404-21) were coated overnight at 4°C with 50 μL of Wuhan-1 RBD (Acrobiosystems SPD-C52H3) (2 µg/mL in PBS). Then, the coating solution was removed, and plates were blocked with 3% non-fat milk in PBS supplemented with 0.1% Tween-20 (P1379-500ML, Sigma) (PBST) for 1 hour at RT. Serum samples were pre-diluted in at 1:50 in 1% non-fat milk in PBST and incubated for 2 hours at RT. After three washes with 250 μL of PBST in a plate washer (Biotek), an anti-human IgG-horseradish peroxidase (HRP)-conjugated secondary antibody (1:5000) (GenScript A01854) was added for 1 hour at RT. Three washes were performed, and after an incubation of 2 min with 100 μL of TMB substrate (Thermo Scientific 34021), the reaction was stopped with 50 μL of stop solution (Thermo Scientific N600). The optical density (OD) was measured at 450 nm in a VictorNivo multimode plate reader (PerkinElmer) and shown OD values were calculated by subtracting the negative control values from all samples.

### Statistics

Statistical analyses were performed with an unpaired two-tailed Student’s t-test. For correlation analyses, Pearson correlation coefficients (r) and their corresponding p-values were calculated. Data were analyzed using Prism 8 (GraphPad). Bland–Altman plots were generated with Graphpad to compare the agreement between nC19BA and the cell-based pseudotyped lentiviral particle assay for the determination of the blocking activity of antibodies.

## Results

### Development of a multiplexed bead array to measure neutralizing activity of antibodies against SARS-CoV-2 variants (nC19BA)

**Figure 1:**
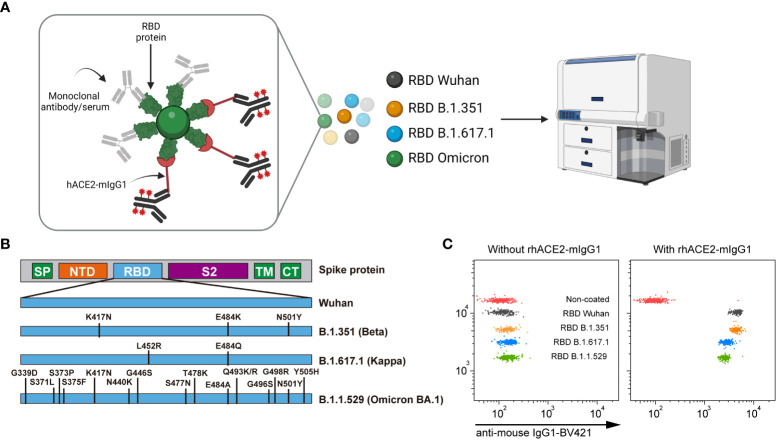
Overview of the SARS-CoV-2 neutralization multiplex assay by flow cytometry (nC19BA). **(A)** Beads with different fluorescence intensities are coated with recombinant RBD proteins from the indicated variants and incubated with a given sample, followed by incubation with rhACE2-mIgG1. The binding of rhACE2 to RBD is detected with an anti-mouse IgG1 monoclonal antibody labelled with BV421 fluorochrome. Schematic created using BioRender.com. **(B)** Schematic illustration showing the mutations present in the RBD variants included in this assay. **(C)** Representative dot plots showing rhACE2 binding to the indicated RBD variants. *SP, Signal peptide; NTD, N-terminal domain; RBD, Receptor binding domain; S2, S2 subunit; TM, transmembrane domain; CT, cytoplasmic tail*.

The nC19BA is a flow cytometry assay that consists of a multiplexed array containing microbeads with different APC fluorescence intensities covalently coated with different RBD variants. The bead array is first incubated with serum samples, allowing the binding of anti-SARS-CoV-2 antibodies to RBD-coated microbeads, followed by the addition of rhACE2-mIgG1. The binding of rhACE2-mIgG1(rhACE2) to RBD-coated microbeads is then measured by flow cytometry after incubation with a fluorescent anti-mouse IgG1 antibody (**Figure 1A**). The neutralizing ability of antibodies is determined by the reduction on the fluorescence signal corresponding to the binding of rhACE2 to RBD-coated microbeads. Selected RBD variants included in this assay are the original RBD (Wuhan-1), the South African B.1.351 (β), the Indian B.1.167.1 (κ) and the South African B.1.1.529 (ο). The mutations present in the RBD of the variants can affect the binding affinity of ACE2. The variants included in nC19BA are shown in **Figure 1B**. We first assessed the ability of this assay to detect the interaction between rhACE2 and different RBD variants. **Figure 1C** shows that rhACE2 binds to RBD-coated microbeads but not negative control beads.

**Figure 2:**
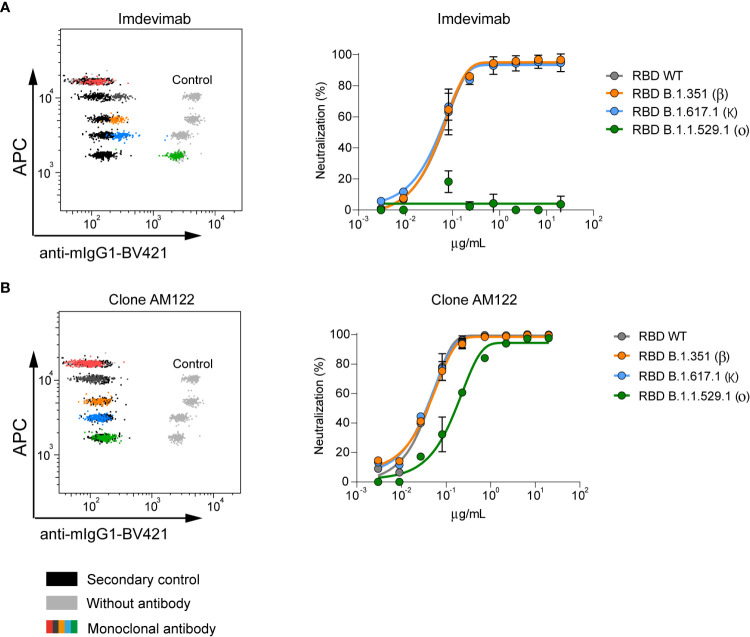
Neutralizing capacity of anti-RBD monoclonal antibodies measured by nC19BA. **(A)** A representative flow cytometry dot plot showing the neutralizing capacity of Imdevimab (25) against the indicated variants (*left*) and a dose-response curve starting at 20 µg/mL (*right*). **(B)** Representative flow cytometry dot plot and dose-response curve of anti-RBD monoclonal antibody, clone AM122 (n=3).

We then interrogated anti-RBD monoclonal antibodies for their ability to block the interaction between rhACE2 and different variants of RBD. To this end, we selected Imdevimab, an anti-RBD therapeutic monoclonal antibody (mAb) that is characterized as a SARS-CoV-2 Wuhan-1 neutralizing antibody without activity against the Omicron BA.1 variant (22–25). These features are clearly observed on **Figure 2A**, which shows that Imdevimab blocks the binding of rhACE2 to all tested variants except Omicron BA.1.

Another anti-RBD mAb, clone AM122, is able to neutralize all tested variants, including Omicron BA.1 although with reduced potency (**Figure 2B**). Together, these initial results indicate that this assay is a promising tool to support therapeutic antibody development with capability to quickly adapt to emerging variants. Importantly, the assay indicates that Omicron BA.1 is a variant that contains mutations that compromise the neutralizing activity of existing antibodies.

### Determination of the neutralizing activity of human serum samples by nC19BA

**Figure 3:**
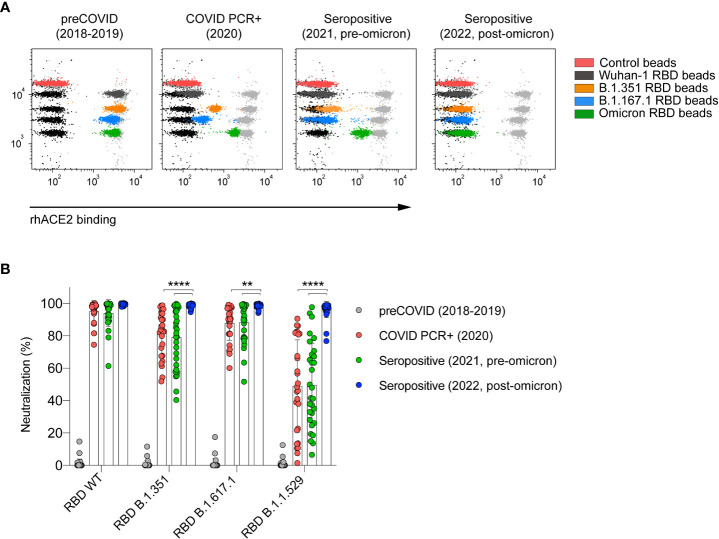
Serum neutralizing activity against SARS-CoV-2 variants depends on the time of sample collection. **(A)** Representative flow cytometry dot plots showing the neutralizing capacity of four serum samples corresponding to the indicated cohorts. **(B)** Comparison of the neutralizing capacity of serums corresponding to the indicated cohorts (n=30 per cohort). Statistical analyses were performed using an unpaired two-tailed Student’s *t*-test. Asterisks represent *p* values (**p < 0.01 and ****p < 0.0001).

In order to determine the ability of nC19BA to measure the neutralizing activity of antibodies at the population level, we selected different cohorts of serum samples based on the time of sample collection, representing infected and/or vaccinated donors at different time-points before and during the pandemic. These cohorts presented different blocking capacity against the tested variants (**Figure 3A**), with significantly reduced ability to neutralize the Omicron BA.1 variant in the case of samples collected before 2022 (**Figure 3B**). In the case of samples collected after the emergence of Omicron BA.1 and the administration of a third dose of the vaccines in early 2022, neutralization of the Omicron BA.1 variant was significantly enhanced and complete, as previously demonstrated by other methods based on cellular infection assays (26–28).

### Correlation between anti-RBD IgG antibody levels and the neutralizing capacity of serum samples

Previous studies showed that there is a strong correlation between serum levels of anti-RBD antibodies and their blocking capacity (29, 30). In this context, we studied the correlation of antibody levels measured by ELISA (**Supplementary table 1**) with the blocking activity against variants measured by the nC19BA flow cytometry assay, employing the same cohorts of seropositive individuals shown in **Figure 3**. Using this method, we observed a significant correlation between anti-RBD (Wuhan-1) antibody levels and their neutralizing activity against the tested variants (**Table 1**). This correlation was weaker in the case of the Omicron BA.1 variant, as a result of the different levels of neutralizing antibodies present in the different cohorts of samples (**Figure 3B**) and their binding capacity to Omicron BA.1 RBD (**Table 1**).

**Table 1:** Correlation analyses between anti-RBD IgG antibody levels present in serum samples and their neutralizing capacity.

RBD variant	n	Pearson correlation (r)	95% confidence interval	R ^(2)^	P value
Wuhan-1	90	0.7134	0.5941 to 0.8020	0.5089	3.004e-15
B.1.351 (β)	90	0.6008	0.4497 to 0.7185	0.361	3.844e-10
B.1.617.1 (κ)	90	0.7275	0.6127 to 0.8122	0.5292	4.649e-16
Omicron BA.1 (ο)	90	0.4031	0.2138 to 0.5632	0.1625	8.183e-05

Correlations between OD values for anti-RBD IgG levels measured by ELISA and their neutralization capacity determined by the nC19BA assay against each of the variants (n=90). Pearson correlation coefficients (r), 95% confidence interval, R squared (R ^(2)^) and their corresponding p-values are shown.

### Comparison of the determination of the neutralizing activity of anti-RBD antibodies by nC19BA and pseudotyped lentiviral particles expressing the spike of SARS-CoV-2

**Figure 4:**
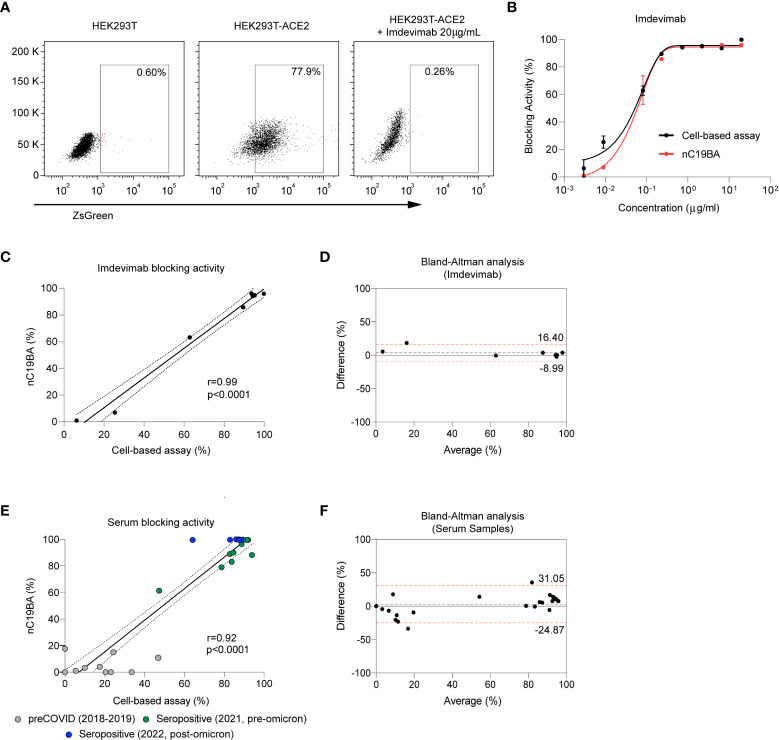
Comparison of the performance of nC19BA against a cell-based neutralization assay. **(A)** Representative dot plots showing the percentage of infected HEK293T (left), HEK293T-ACE2 (middle) and HEK293T-ACE2 cells in the presence of Imdevimab (right), after incubation with pseudotyped viral particles expressing the Spike of SARS-CoV-2, measured by flow cytometry. **(B)** Comparison of the blocking activity achieved by the indicated concentrations of Imdevimab measured by two independent methods: a cell-based pseudotyped lentiviral assay (black) or the bead-based nC19BA assay (red). **(C)** Pearson correlation corresponding to the blocking activity of Imdevimab measured by nC19BA and a cell-based pseudotyped lentiviral assay. Pearson correlation coefficient (r), 95% confidence interval and its corresponding p-values are shown. **(D)** Bland-Altman analysis showing the agreement between nC19BA and a cell-based pseudotyped lentiviral assay for the measurement of the blocking activity of Imdevimab. **(E)** Pearson correlation corresponding to the blocking activity of preCOVID (n=10) and seropositive (n=18) serum samples corresponding to the indicating cohorts, measured by nC19BA and a cell-based pseudotyped lentiviral assay. Pearson correlation coefficient (r), 95% confidence interval and its corresponding p-values are shown. **(F)** Bland-Altman analysis showing the agreement between nC19BA and a cell-based pseudotyped lentiviral assay for the measurement of the blocking activity of serum samples.

In order to compare the performance of the bead-based neutralization assay with established surrogate methods, we measured the neutralizing activity of Imdevimab against the Wuhan-1 strain in both assays. **Figure 4A** shows that pseudotyped lentiviral particles expressing the spike of SARS-CoV-2 and encoding for a fluorescent reporter readily infect 293 cells overexpressing ACE2, and infection is effectively blocked by Imdevimad, as expected (31). The blocking activity percentages obtained in a dose response curve in this cellular assay were comparable to those obtained by the bead-based assay (**Figure 4B**), showing a strong correlation (**Figure 4C**). A Brand-Altman analysis demonstrated agreement between both methods (**Figure 4D**).

We then compared the performance of bead-based and cell-based assays for the determination of the neutralizing capacity of serum samples corresponding to preCOVID (n=10) and seropositive (n=18) cohorts. **Figure 4E** shows correlation in the neutralizing percentage assessed by both methods, and a Brand-Altman analysis was performed to demonstrate agreement (**Figure 4F**).

## Discussion

Neutralizing antibodies play a critical role as a barrier of defense against viral infections (32), and their measurement is a good surrogate marker to determine immune protection against SARS-CoV-2 (33). Several strategies to measure the levels of SARS-CoV-2 specific antibodies in biological samples have been developed (18, 34). The emergence of variants with the ability of evading previous humoral immunity as a result of RBD mutations has resulted in an ongoing need for developing assays that measure the neutralizing activity of antibodies. Neutralizing assays have relevant applications in drug discovery, vaccination strategies and pandemic preparedness.

Current assays to measure the neutralizing activity of antibodies are based on the evaluation of viral infection of cell cultures, either using viral isolates or pseudotyped lentiviral particles expressing the spike of SARS-CoV-2 (21, 35–37). Logistic and scalability limitations of cell-based assays include time, safety, reproducibility, cost and sample volume (38). Bead-based assays offer an alternative that overcomes these barriers. In this context, novel methodologies have been developed or adapted, but require the measurement of fluorescence in closed systems (i.e., Luminex) (39, 40). The assay presented here (nC19BA) allows the evaluation of the blocking activity of monoclonal antibodies or serum samples against multiple SARS-CoV-2 variants simultaneously, based on the measurement of the ACE2-RBD interaction by flow cytometry. In this context, a limitation of the presented bead-based assay is the inability to detect blocking antibodies that bind to regions of the Spike other than the RBD (7, 8).The modular nature of this assay can be adapted to include other emerging variants or antigens that can be easily coupled to additional beads, leveraging the flexibility offered by the biotin-streptavidin binding method (18).

This assay can support drug discovery efforts. An example is antibody discovery in the context of neutralization of emerging variants. We show that this assay can be used to interrogate antibodies for this application by assessing Imdevimab, a monoclonal antibody approved for the treatment of COVID-19 (41, 42). Imdevimab blocks the interaction between rhACE2 and RBD (Wuhan-1), but this effect is completely evaded by the Omicron BA.1 variant as demonstrated here in agreement with other studies using established methods to assess neutralization (i.e. cell-based assays using SARS-CoV-2 pseudotyped viral particles) (22–25). Given the medium to high-throughput capability of flow cytometry (43–45), nC19BA is a suitable assay for performing antibody discovery campaigns.

The nC19BA assay also has relevant applications in the analyses of serum samples and their neutralizing activity. By using this method, we show that samples collected after the deployment of the third dose of the vaccine have substantially increased neutralizing capacity against Omicron BA.1, as expected (46–48). In this context, this assay could support the development of new vaccines, the prediction of their efficacy against emerging variants, and the rational prioritization of individuals to receive additional doses.

We compared the performance of nC19BA to a cell-based pseudotyped lentiviral neutralization assay, demonstrating agreement between both assays for the evaluation of the neutralizing capacity of monoclonal antibodies and serum samples. These comparable results are of relevance given that the proposed bead-based assay assesses the blockade of the ACE2-RBD interaction, while the cell-based assay interrogates infection by pseudotyped lentiviral particles as a genuine surrogate method to assess neutralizing activity.

In summary, we have developed a cell-free flow cytometry assay that determines the neutralizing activity of monoclonal antibodies or serum samples against four SARS-CoV-2 variants and could be easily adapted to include future variants.

## Data availability statement

The original contributions presented in the study are included in the article/**Supplementary Material**. Further inquiries can be directed to the corresponding author.

## Ethics statement

The studies involving human participants were reviewed and approved by Basque Clinical Research Ethics Committee and Cantabria Ethics Committee. The patients/participants provided their written informed consent to participate in this study.

## Author contributions

Conceptualization: LE-M and AP. Methodology: LE-M, ABo, EP-F, LV-V, SZ, SL, BJ-L, AG, AA-V, ABl, PV-B, MM, EG and SB. Sample sourcing: MS-M, PI, NE, MM-C, ML-H, JM and OM. Formal analysis: LE-M and ABo. Writing original draft: LE-M and AP. Review and editing: All authors. All authors contributed to the article and approved the submitted version.

## Funding

This research was supported by the SPRI I+D COVID-19 fund (Basque Government, bG-COVID-19), BIOEF EITB Maratoia (BIO21/COV/037 to AP), the European Research Council (ERC) (ERC-2018-StG 804236-NEXTGEN-IO to AP), the Instituto de Salud Carlos iii (ISCiii, DTS21/00094 to AP and DTS20/00138 to MM-C), Ministerio de Ciencia, Innovación y Universidades (MICINN, PID2019-107956RA-I00 and TED2021-129433B-C21 to AP; PID2020-117116RB-I00 and RTC2019-007125-1 to MM-C) and the FERO Foundation to AP. Personal fellowships: EP-F (Juan de la Cierva-Formación, FJC2018-035449-I), ABo (AECC Bizkaia Scientific Foundation, PRDVZ19003BOSC), AG (Programa Bikaintek from the Basque Government, 48-AF-W1-2019-00012), AA-V (La Caixa Inphinit, LCF/BQ/DR20/11790022), BJ-L (Basque Government, PRE_2019_1_0320), ABl (AECC Bizkaia Scientific Foundation, PRDVZ21640DEBL), PV-B (Proyectos I+D+I, PRE2020-092342) and AP (Ramón y Cajal, RYC2018-024183-I; and Ikerbasque Research Associate).

## Acknowledgments

The plasmids for the generation of pseudotyped lentiviral particles were kindly provided by Dr Jesse D. Bloom (Fred Hutchinson Cancer Research Center) and Dr Jean-Philippe Julien (The Hospital for Sick Children). HEK293T-ACE2 cells were kindly provided by Dr. June Ereño-Orbea (CIC bioGUNE) and Dr. Jean-Philippe Julien (The Hospital for Sick Children Research Institute, Toronto).

## Conflict of interest

MM, EG and SB are employees of and hold stock in BD Biosciences.

The remaining authors declare that the research was conducted in the absence of any commercial or financial relationships that could be constructed as a potential conflict of interest.

## Publisher’s note

All claims expressed in this article are solely those of the authors and do not necessarily represent those of their affiliated organizations, or those of the publisher, the editors and the reviewers. Any product that may be evaluated in this article, or claim that may be made by its manufacturer, is not guaranteed or endorsed by the publisher.
